# Where Are We With Teledermatology? Two Years in the Wake of COVID-19

**DOI:** 10.2196/47168

**Published:** 2023-07-06

**Authors:** Wilson Sim, Ellie Choi, Nisha Suyien Chandran

**Affiliations:** 1 Yong Loo Lin School of Medicine National University of Singapore Singapore Singapore; 2 Division of Dermatology Department of Medicine National University Hospital Singapore Singapore

**Keywords:** teledermatology, telehealth, teleconsultation, COVID-19, dermatology, trends, sustainability, uptake, telemedicine, global trend, low quality of care, technological barrier, virtual care

## Introduction

Much attention was brought to teledermatology during the COVID-19 pandemic, but how has this trend held up? Two years on, we review the trends of teledermatology in our institute and globally, and discuss the role of teledermatology moving forward.

## Methods

Outpatient live video teleconsults planned and conducted in the National University Hospital between January 2020 to December 2022 were retrospectively extracted. Planned teleconsults were defined as those scheduled by doctors for a future date (typically between 1 week to 1 year). Teleconsults conducted refer to those that had actualized. These numbers were presented as percentages of total outpatient consultations (teleconsults and physical). In our setting, teleconsults are typically planned for patients on chronic follow-up who do not require in-clinic procedures, regardless of diagnosis and disease severity. All new referrals and first visits were seen in person. Reasons for patients declining a planned teleconsult were collected and tabulated.

## Results

After the initial uptake of teledermatology in Q2 2020 secondary to the COVID-19 pandemic, the percentage of teleconsults conducted remained largely stable between 1% and 2%. In contrast, the percentage of teleconsults planned showed fluctuations, increasing from 0% in Q1 2020 to 6.3% in Q4 2020, decreasing to 3% in Q1 2021 before increasing again to 4.3% in Q3 2021.

**Figure 1:**
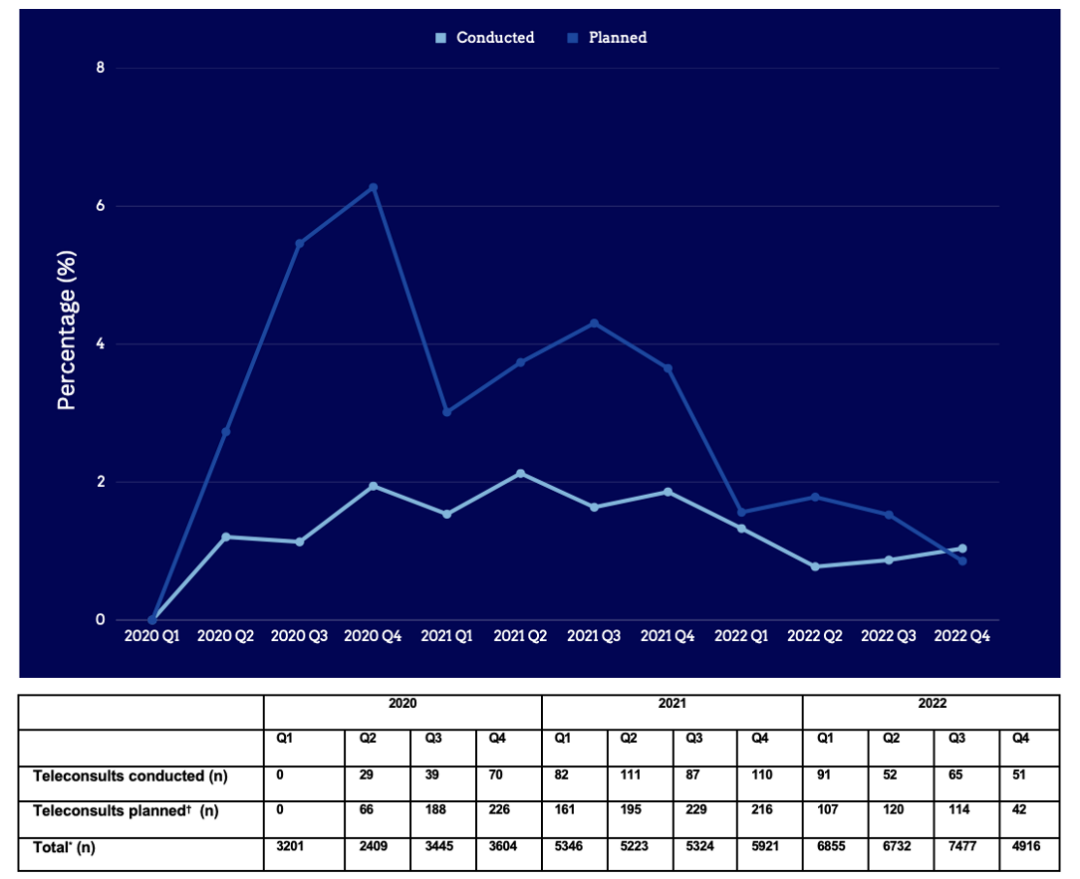
Percentage of conducted and planned teleconsults of total outpatient consultations. In Q2 2020, a lockdown in Singapore (termed "Circuit Breaker") limited movement outside the house to essential activities, and nonurgent outpatient services were advised to be postponed or switched to teleconsult. In the following quarters of Q3 and Q4 2020, outpatient services were prioritized based on necessity and capacity. From 2021 Q1 onward, a "Safe Nation" approach allowed for the full resumption of outpatient services, businesses, and reopening of borders. *Total refers to the combined number of teleconsults and physical consultations.

Subsequently, the percentage of teleconsults planned decreased again to 0.9% in Q4 2022 (Figure 1). These increases in teleconsults planned over the 2 years corresponded with institutional efforts to promote teledermatology through visual (clinic posters) and verbal prompts (weekly department meetings). Clinic service assistants were also trained to schedule and manage video teleconsults including setting up, alerting patients, and managing waiting rooms to facilitate the transition between physical and virtual consultations and to improve patient experience.

Reasons cited by patients for declining a proposed teleconsult included having to pay the same price as physical consults (n=80) and a perceived lower quality of care (n=79), which they worry could lead to misdiagnosis or inadequate management of flares. Technological barriers (n=49) such as difficulty in setting up videoconferencing applications are another commonly cited reason.

## Discussion

The stable percentage of teleconsults conducted despite institutional and physician efforts to promote teleconsultations is interesting. It may reflect a small but stable population of patients keen to do teleconsultation, with the remaining that are harder to convince for reasons such as perceived lower quality, lack of financial incentives, and technological barriers.

Our findings indicate a lower uptake of teledermatology compared to global reports [1], where for example, 50% of patients with acne and 72% of patients with atopic dermatitis preferred teledermatology [2]. Besides the reasons shared by patients for declining proposed teleconsults, additional contextual factors may be involved. In our setting, patients still had the option of physical consultation or to postpone nonurgent consultations even during lockdown periods. Physical accessibility is also seldom an issue in the city-state of Singapore; thus, the time and cost savings from transportation for medical care are marginal. Furthermore, consulting a dermatologist physically is financially accessible to most Singaporeans, and teledermatology is therefore not used as a means to access care (as opposed to some underserved areas in the United States where teledermatology through a store-and-forward system may be the only affordable avenue for patients to access dermatological consult) [3,4].

Then, is there a role of teledermatology in areas like Singapore? We believe there is. First, we should recognize the potential need to pivot rapidly from physical to virtual consultation due to pandemics, natural disasters, armed conflicts, or other mass casualty events. Second, the inclusion of teledermatology during peacetime provides an additional service option that increases patient satisfaction especially if it is seamless, secure, and easy to use [5]. It also offers a glimpse into the possible futures of health care delivery such as in the metaverse.

The pandemic has shown that teledermatology can be an effective and feasible model of care; however, the suitability of teledermatology may be individual specific. The willingness to teleconsult is influenced by patient factors such as the patient’s disease perception and the purpose of the consult [6,7]. For example, teledermatology was more acceptable to patients with stable and mild diseases [1,7]. Other patient factors include younger age, higher perceived accuracy of teledermatology, and increased willingness to show sensitive body areas, which is correlated with increased security of teleconsult platforms [5,7,8]. Teledermatology may also help primary care physicians triage patients, expedite and facilitate timely referrals, and has also been shown to reduce face-to-face specialist appointments leading to cost-effective dermatological care [5,9,10].
